# Knowledge, attitude, and practice of Egyptian medical students towards healthcare workers’ recommended vaccines: a nationwide cross-sectional survey

**DOI:** 10.1186/s12909-024-05712-8

**Published:** 2024-08-14

**Authors:** Mohamed Mohamed Shawqi, Yara Mohamed El-Said, Mostafa B. Behery, Ali Abdelaziz, Esraa Shawky Ibrahem, Aly ElBoraie, Mohamed Ayman Khattab, Ahmad S. Ghattas, Ahmed Naeem, Maysa Madany, Toka Elboraay, Mostafa Mahmoud Naguib, Abdallah R. Allam, Ahmed Hafez Allam, Ammar Ayman Bahbah, Marwa Ibrahim Ewis, Mostafa Ahmed Elsayed, Leenah Sherief, Mahmoud Tawfik KhallafAllah, Mohamed Alaa Gouda, Omar Ali Aboshady

**Affiliations:** 1 Department of Emergency Medicine, Faculty of Medicine, Benha University, Benha, Egypt; 2 Student Research Unit, Faculty of Medicine, Menoufia University, Menoufia, Egypt; 3 Faculty of Medicine, Alexandria University, Alexandria, Egypt; 4 Faculty of Medicine, Cairo University, Cairo, Egypt; 5 Faculty of Medicine, Helwan University, Helwan, Egypt; 6 Faculty of Medicine, Al-Azhar University, Assiut, Egypt; 7 Faculty of Medicine, South Valley University, Qena, Egypt; 8 Faculty of Medicine, Zagazig University, Zagazig, Egypt; 9 Faculty of Medicine, Al-Azhar University, Damietta, Egypt; 10 Department of ophthalmology, Faculty of Medicine, Menoufia University, Menoufia, Egypt; 11 Department of Clinical Oncology, Faculty of Medicine, Menoufia University, Menoufia, Egypt; 12 Clinical Pharmacology Department, Faculty of Medicine, Menoufia University, Menoufia, Egypt

**Keywords:** Healthcare worker, Booster, Vaccine, Egypt, Medical student, COVID-19, Flu vaccine

## Abstract

**Background:**

Vaccination of healthcare workers (HCWs) is pivotal in decreasing the incidence of contagious infections in hospital settings. In this study, we assessed the knowledge, attitude, and practice regarding HCWs’ recommended vaccines among medical students and interns in Egypt.

**Methods:**

A multicenter, cross-sectional study was conducted using a structured, pilot-tested, and self-administered questionnaire among Egyptian medical students and interns. We invited 1332 participants to our survey using a systematic random sampling that included participants across nine medical schools in Egypt during the 2021–2022 academic year.

**Results:**

Out of 1332 participants, 1141 completed our questionnaire with a response rate of 85.7%. Overall, 43% of the participants had intermediate knowledge (knew 2–3 HCWs’ recommended vaccines). Furthermore, 36.7% had received a booster dose of at least one of the HCWs’ recommended vaccines over the last 10 years, with only 6.1% having received all recommended vaccines. Hepatitis B vaccine was the most widely known (71%) and received (66.7%). Interns were more likely to know, receive, and recommend HCWs’ recommended vaccines. The majority (> 90%) agreed that vaccination is beneficial and safe, with a median score of eight (interquartile range [IQR: Q25-Q75]: 7–9) out of ten for vaccine efficacy and eight (IQR: 7–8) for safety. However, the median score for hesitancy was five (IQR: 2–7). The most common influential and limiting factors for vaccination were scientific facts (60.1%) and fear of vaccine side effects (44.9%).

**Conclusion:**

Although medical students in Egypt have good knowledge of and attitudes towards vaccination, there is a gap in their practices. Interventions are needed to improve vaccination uptake among medical students in Egypt.

**Supplementary Information:**

The online version contains supplementary material available at 10.1186/s12909-024-05712-8.

## Introduction

Vaccination plays a crucial role in preventing infections in communities and healthcare settings, particularly among high-risk individuals. World Health Organization (WHO) and Center for Disease Control (CDC) prioritize vaccination coverage for children and high-risk groups including healthcare workers (HCWs) [1]. The CDC strongly recommends that HCWs receive vaccines such as Hepatitis B, MMR (Measles, Mumps, Rubella), Varicella (Chickenpox), Influenza, and DTaP (Diphtheria, Tetanus, Pertussis) [1, 2]. Moreover, it recommends that microbiologists and HCWs who come into contact with Neisseria meningitidis-infected individuals should receive meningococcal vaccine [1]. Additionally, due to the gap between childhood vaccinations and working as an HCW, booster doses or revaccination may be necessary to maintain high levels of immunity.

Ensuring high vaccination rates among HCWs is critical to prevent the transmission of infections to patients and maintain the integrity of the healthcare system. This is particularly relevant in the context of the COVID-19 pandemic, which highlights the importance of vaccination in preventing the spread of COVID-19 [3, 4]. However, there is an increasing rate of vaccine hesitancy, not only in general population but also among HCWs which has impacted vaccine-related behavior and uptake [5–7]. This is concerning as HCWs are considered to be the most influential for patients regarding vaccinations [8]. Patient decision about vaccination is usually associated with their trust in their HCWs [9].

Medical students are the future of the healthcare system and their attitude and practice towards vaccines will shape the future of vaccine uptake among HCWs and patients. Internationally, multiple studies have evaluated the knowledge and attitude of medical students towards vaccines. However, most of these studies included only one or a limited number of vaccines, such as influenza, HBV, and COVID-19 [10–12]. Generally, medical students are aware of the importance of vaccination, however there is a gap between knowledge and practice. Rostkwoska et al. reported that 99.2% of European medical students were aware of booster vaccinations; however, only 68% had taken any [13], which could be attributed to vaccine hesitancy.

In low- and middle-income countries, it is estimated that vaccination coverage for HCWs is lower than high-income countries. For example, WHO has estimated that only 18–39% of HCWs in low- and middle-income countries have HBV vaccination coverage compared to 67–79% in high-income countries [14]. In Egypt, most of the available studies have evaluated the attitudes of medical students towards only COVID-19 vaccination. Saied et al. explored the beliefs and barriers of medical students towards COVID-19 vaccines and found that 46% of Egyptian medical students were hesitant to receive COVID-19 vaccines [15]. Another study evaluated vaccination rates and found that 83.2% of Egyptian medical students were vaccinated [16]. However, to our knowledge, there is no or limited data regarding the other HCWs’ recommended vaccines.

This study is underpinned by the Health Belief Model (HBM) and the Theory of Planned Behavior (TPB), which provide a theoretical basis for understanding health behaviors, including vaccination uptake [17]. The HBM posits that individuals are more likely to engage in health-promoting behaviors if they perceive a higher susceptibility to a health issue, believe the health issue has serious consequences, think taking a specific action would reduce their susceptibility or severity, and perceive fewer barriers to taking that action [18]. The TPB, on the other hand, suggests that an individual’s behavior is influenced by their intentions, which are shaped by their attitudes towards the behavior, subjective norms, and perceived behavioral control [19]. Understanding the current knowledge and attitude of medical students and interns towards HCWs’ recommended vaccines will help to develop strategies to improve vaccination rates and protect the health of both HCWs and the population.

This study aims to assess the prevalence, knowledge, and attitudes towards HCWs’ recommended vaccinations among medical students and interns in Egypt. Furthermore, being shortly after the pandemic, it has special focus on COVID-19 and flu vaccines as they were highly recommended. Additionally, it examines the factors influencing the use of booster doses.

The specific research questions are:

1. What is the prevalence of uptake of HCWs’ recommended vaccinations among medical students and interns in Egypt?
2. What is the level of knowledge and what are the attitudes of medical students and interns towards these vaccinations?
3. What factors influence the uptake of booster doses among medical students and interns?

## Materials and methods

We conducted a multicenter, cross-sectional study using a structured, pilot-tested, and self-administered questionnaire to evaluate the knowledge, attitude, and practice of medical students regarding HCWs’ recommended vaccines. This study was approved by the institutional review board of Benha University.

### Study population and sample size

Our population included undergraduate medical students and interns from Egyptian universities during the 2021–2022 academic year, regardless of nationality. Universities that recently opened without students in all study years were excluded. Of the 26 eligible Egyptian universities in the seven regions, 10 were randomly selected using stratified random sampling based on the number of eligible universities in each region. The selected universities were Cairo University, Benha University, Helwan University (Cairo region), Alexandria University (Alexandria region), Menoufia University, Damietta Branch of Al-Azhar University (Delta region), Zagazig University (Canal region), South Valley University (South Upper Egypt region), Assiut Branch of Al-Azhar University (Middle-Upper Egypt region), and Fayoum University, which were later excluded due to communication difficulties.

Using Raosoft online calculator (https://www.raosoft.com/samplesize.html), we calculated the sample size to achieve a 99% confidence interval, a 4% margin of error, and a 50% response rate to increase the power and accuracy to capture the diversity across medical universities throughout Egypt. This yielded a sample size of 1024. To account for a 30% non-response rate, the total required sample was 1332 which was divided proportionately among universities based on the overall number of students. Within each university, we used a simple random sampling technique, and the sample was divided equally between academic, clinical, and internship years. The first three years were considered academic, the next three years were clinical, and the last year was the internship year. We randomly selected participants from the students’ lists for each class using random.org.

### Data collection

University teams were formed with local coordinators, who obtained student lists from official sources. Invitations to participate were sent to the selected individuals between September and November 2022 via email and social media platforms, with a unique code provided to each participant. A voluntary consent form was required for participation.

### Questionnaire development

The questionnaire was developed by the principal investigators through a literature review and reviewed by two experts for clarity and relevance. A pilot study was conducted among seven universities to gather feedback on the questionnaire’s format, clarity, and completion time. Changes were made based on this feedback to improve question clarity. Pilot study responses were not included in the final analysis. The questionnaire, consisting of 29 questions divided into four sections, was administered in English using Google Forms. It included a cover letter, sociodemographic questions, practice and knowledge questions, attitudes towards vaccination questions, and attitudes towards specific vaccines (COVID-19 and flu). The questionnaire can be found in the supplementary files.

### Statistical analysis

Descriptive statistics are presented as numbers and percentages for qualitative data and median and interquartile range (IQR) [Q25-Q75] for quantitative data. We used the chi-square test to assess the associations between categorical variables. Wilcoxon rank sum test (Mann Whitney) and Kruskal Wallis test were used to assess the association between gender, educational level, and attitudes regarding efficacy, safety, and hesitancy. Knowledge about recommended vaccines was divided into three categories: low knowledge (0–1 vaccines), intermediate knowledge (2–3 vaccines), and high knowledge (4–6 vaccines). A p-value of 0.05 was used as the limit for statistical significance. Statistical analyses were performed using the SAS statistical software (version 9.4; SAS Institute Inc., Cary, NC, USA).

## Results

We collected data from nine of the ten selected universities. A total of 1,321 individuals were randomly selected from nine universities located across six regions of Egypt. Of these, 1,141 students completed the survey, resulting in a response rate of 86.4%. The demographic characteristics of the participants are summarized in Table 1. Most of the respondents were Egyptian (89.4%), male (51.9%), first-generation medical students (65.6%), and living in urban areas (63.6%). Furthermore, 95% of the participants had received vaccinations as part of a national campaign during infancy, and 78% reported having access to a vaccination center at their institution or governorate.

**Table 1:** Sociodemographic characteristics of the participants

Variables	Number (%)
**Gender**	
Male	592 (51.9%)
Female	549 (48.1%)
**Educational level**	
Academic	404 (35.4%)
Clinical	391 (34.3%)
Intern	346 (30.3%)
**Region**	
Cairo Region	447 (39.2%)
Alexandria Region	261 (22.9%)
Delta Region	178 (15.6%)
Canal Region	159 (13.9%)
Assuit Region	46 (4.0%)
South Upper Egypt Region	50 (4.4%)
**First generation**	
Yes	748 (65.6%)
No	393 (34.4%)
**Current residency**	
Rural	415 (36.4%)
Urban	726 (63.6%)
**Nationality**	
Egyptian	1020 (89.4%)
Non-Egyptian	121 (10.6%)
**Vaccination during infancy**	
Yes	1089 (95.8%)
No	12 (1.1%)
Don’t know	40 (3.5%)
**Availability of local vaccination center**	
Yes	890 (78%)
No	50 (4.4%)
Don’t know	201 (17.6%)

### Knowledge of booster vaccinations

**Fig. 1:**
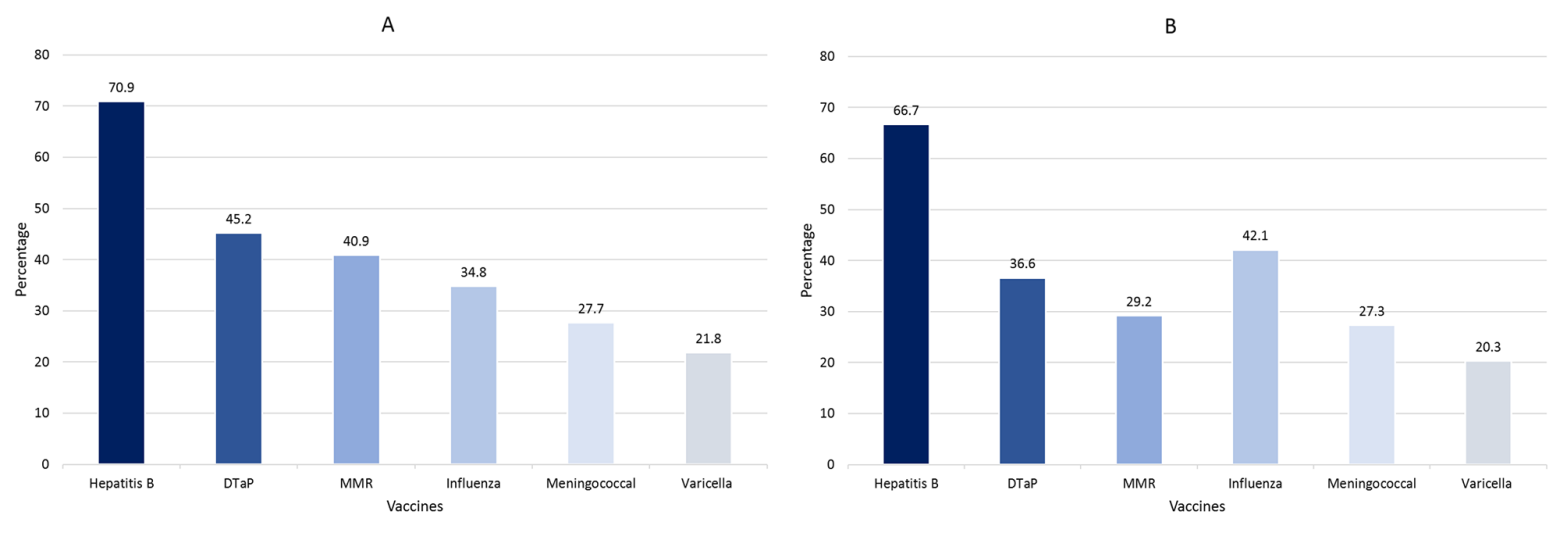
Knowledge (**A**) and prevalence (**B**) of different HCWs’ recommended vaccines

Approximately 85% of participants reported knowing at least one HCW-recommended vaccine, while only 3.8% were knowledgeable about all six recommended vaccines. Moreover, 43% and 25% of the participants had intermediate and high knowledge, respectively (Table 2). The most commonly recognized vaccine was HBV vaccine (Fig. 1A). Interns and clinical-year students were more likely to have intermediate knowledge than academic-year students (49.7% and 49.1% vs. 32.4%, *P* < 0.001). Moreover, males were more likely to have higher knowledge than females (27.7% vs. 22.6%, *P* = 0.003) and were more likely to have intermediate knowledge (Table 2). Additionally, Egyptian nationality (44.6% vs. 33.1% for non-Egyptians, *P* = 0.0457) and geographical region (*P* = 0.0329) significantly influenced intermediate knowledge. Other sociodemographic factors, including type of residence and generation, were not significantly associated with knowledge (*P* > 0.05).

**Table 2:** Knowledge of HCWs’ recommended vaccines among medical students and interns

Knowledge levels	Total	Gender		*p*	Educational level			*p*
		Male	Female		Academic	Clinical	Interns	
**Low knowledge**	358 (31.4%)	200 (33.8%)	158 (28.8%)	**0.003**	164 (40.6%)	102 (26.1%)	92 (26.6%)	**0.001**
**Intermediate knowledge**	495 (43.4%)	228 (38.5%)	267 (48.6%)		131 (32.4%)	192 (49.1%)	172 (49.7%)	
**High knowledge**	288 (25.2%)	164 (27.7%)	124 (22.6%)		109 (27.0%)	97 (24.8%)	82 (23.7%)	

### Booster vaccination practice

About 36.7% of the participants reported receiving booster doses of at least one HCW-recommended over the past ten years. However, a small proportion (6.1%) reported receiving all recommended vaccine boosters, whereas 8.1% reported receiving 2–3 vaccine boosters. The most commonly reported vaccines among those who received booster doses were HBV (66.7%) and influenza (42.1%) (Fig. 1B). Educational grade (interns: 56.1% vs. academic: 30% vs. clinical: 26.3%; *P* < 0.0001), and geographical region (South Upper region: 56%; *P* = 0.0153) were significantly associated with higher prevalence. Interestingly, the presence of a vaccination center was significantly associated with receiving at least one HCW-recommended vaccine (yes: 39.3%, no: 28%, not sure: 26.9%; *P* = 0.0018). No significant associations were found for gender, residence, nationality, or generation (*P* > 0.05).

### Attitudes towards booster vaccination

More than 90% of the participants agreed that vaccination is useful and safe and that everyone should be vaccinated. Additionally, 92.2% of participants recognized the necessity of booster doses to ensure adequate protection (Table 3). Interns demonstrated higher awareness of revaccination importance and compliance (37%) compared to clinical and academic students (28.1% and 31.7%, respectively; *P* < 0.001). Conversely, clinical students exhibited awareness but uncertainty regarding full vaccination, while academic students were more likely to encounter this information for the first time compared to interns and clinical students. Type of residence (urban: 34.4% vs. rural: 28%; *P* = 0.0116), presence of a vaccination center (yes: 34.7%, no: 22%, not sure: 22.9%; *P* = 0.0038), and nationality (Egyptian: 31% vs. non-Egyptians: 41.3%; *P* = 0.0009) were also significantly associated with awareness of the need for revaccination. Interestingly, over 50% of the participants reported an intention to take booster doses or at least perform a blood analysis to ensure immunity.

**Table 3:** Attitudes of medical students and interns towards HCWs’ recommended vaccines

Attitude questions	Total	Gender		*p*	Educational level			*p*
		Male	Female		Academic	Clinical	Interns	
**In general, which statement describes your opinion about vaccinations?**								
It is useful and safe, and I think that everybody should get vaccinated.	1048 (91.8%)	546 (92.2%)	502 (91.4%)	0.60	360 (89.1%)	364 (93.1%)	324 (93.6%)	0.21
There is too little evidence to prove that it is effective.	50 (4.4%)	22 (3.7%)	28 (5.1%)		25 (6.2%)	13 (3.3%)	12 (3.5%)	
There is too little evidence to prove that it is safe.	37 (3.2%)	20 (3.4%)	17 (3.1%)		15 (3.7%)	13 (3.3%)	9 (2.6%)	
It is neither effective nor safe.	6 (0.5%)	4 (0.7%)	2 (0.4%)		4 (1%)	1 (0.3%)	1 (0.3%)	
**Do you know that in order to be protected properly you need to get revaccinated for several vaccines?**								
Yes, I am aware it and doing it properly.	366 (32.1%)	209 (35.3%)	157 (28.6%)	0.85	128 (31.7%)	110 (28.1%)	128 (37%)	**0.001**
Yes, I am aware of it, but I am not sure if I have full vaccination.	686 (60.1%)	337 (56.9%)	349 (63.6%)		224 (55.4%)	256 (65.5%)	206 (59.5%)	
No, this is the first time I hear about that.	63 (5.5%)	31 (5.5%)	32 (5.8%)		37 (9.2%)	17 (4.3%)	9 (2.6%)	
No, there is no need because vaccination is always life-long protection.	26 (2.3%)	15 (2.5%)	11 (2%)		15 (3.7%)	8 (2%)	3 (0.9%)	
**Do you intend to take booster doses of HCWs recommended vaccines or have a blood test to ensure immunity?**								
Yes	602 (52.8%)	310 (52.4%)	292 (53.2%)	0.42	186 (46%)	209 (53.5%)	207 (59.8%)	**0.001**
No	146 (12.8%)	83 (14%)	63 (11.5%)		50 (12.4%)	49 (12.5%)	47 (13.6%)	
Not sure	393 (34.4%)	199 (33.6%)	194 (35.3%)		168 (41.6%)	133 (34%)	92 (26.6%)	
**Do you think that booster doses for health care workers’ recommended vaccines should be mandatory for medical stuff? (Attending doctors, nurses, etc.)**								
Yes	1002 (87.8%)	507 (85.6%)	495 (90.2%)	0.06	347 (85.9%)	341 (87.2%)	314 (90.8%)	0.09
No	56 (4.9%)	36 (6.1%)	20 (3.6%)		21 (5.2%)	17 (4.3%)	18 (5.2%)	
Not sure	83 (7.3%)	49 (8.3%)	34 (6.2%)		36 (8.9%)	33 (8.4%)	14 (4%)	
**Do you think that booster doses for health care workers’ recommended vaccines should be mandatory for medical students?**								
Yes	766 (67.1%)	376 (63.5%)	390 (71%)	**0.002**	249 (61.6%)	268 (68.5%)	249 (72%)	**0.01**
No	170 (14.9%)	109 (18.4%)	61 (11.1%)		61 (15.1%)	58 (14.8%)	51 (14.7%)	
Not sure	205 (18%)	107 (18.1%)	98 (17.9%)		94 (23.3%)	65 (16.6%)	46 (13.3%)	
**Do you recommend your relatives, friends, colleagues etc. to take a booster dose of vaccines?**								
Yes	843 (73.9%)	435 (73.5%)	408 (74.3%)	0.12	277 (68.6%)	282 (72.1%)	284 (82.1%)	**0.001**
No	64 (5.6%)	41 (6.9%)	23 (4.2%)		26 (6.4%)	19 (4.9%)	19 (5.5%)	
Not sure	234 (20.5%)	116 (19.6%)	118 (21.5%)		101 (25%)	90 (23%)	43 (12.4%)	

Regarding the mandatory nature of booster vaccination, approximately 88% and 67% of the participants believed that these vaccines should be mandatory for medical staff and medical students, respectively. Interns were more likely to support mandatory vaccination than academic students (72% vs. 62.6%, *P* = 0.01). Additionally, females were more likely than males to endorse mandatory vaccination for medical students (71% vs. 63.5%, *P* = 0.002). Furthermore, 74% of participants indicated that they would advise their colleagues, friends, and family to receive booster vaccinations. Educational grade (Table 3) and type of residence (urban: 75.6% vs. rural: 70.8%; *P* = 0.044) were significantly associated with the intention to recommend booster vaccination. Moreover, the presence of a vaccination center at the participants’ institutions or governorates was significantly associated with the intention to recommend booster vaccination (yes: 38%, no: 15.6%, not sure: 15.4%; *P* = 0.0008). However, other sociodemographic characteristics, including gender, region, generation, and nationality, were not associated with this recommendation (*P* > 0.05).

Out of ten, our participants reported a median score of eight (IQR: 7–9) for efficacy, a median score of eight (IQR: 7–8) for safety, and a median score of five (IQR: 2–7) for hesitancy. Regarding efficacy, interns (median [IQR] score = 8 [7–9]) and clinical students (median [IQR] score = 8 [7–9]) were more likely (*P* < 0.0001) to have higher median scores than academic students (median [IQR] score = 7 [6–8]). In addition, Egyptian students had higher median efficacy scores than non-Egyptian students (median [IQR] = 8 [7–9] vs. 7 [6–8]; *P* < 0.0001). The presence of a vaccination center was also associated with higher efficacy scores (median [IQR] score = 8 [7–9] vs. 7 [5–8] for no; *P* < 0.0001).

Regarding safety, males (median [IQR] score = 8 [7–9] vs. 8 [7–8] for females; *P* = 0.0095), Egyptian students (median [IQR] score = 8 [7–9] vs. 7 [6–8] for non-Egyptians; *P* = 0.0157), and interns and clinical-year students (median [IQR] score = 8 [7–9] and 8 [7–9] vs. 8 [6–8] for academic-year; *P* < 0.0001] were more likely to have higher median scores (Table 4). The presence of a vaccination center was also associated with higher median scores (median [IQR] score = 8 [7–9] vs. 7 [6–8]; *P* = 0.005] for safety. Neither the type of residence nor region was associated with safety or efficacy (*P* > 0.05). Additionally, no significant association was found with hesitancy in any of the demographic data.

**Table 4:** Median, interquartile range and mean rank values of scores assessing safety, efficacy, and hesitancy of HCWs’ recommended booster vaccination

Score	Total	Gender*		*p*	Educational level**			*p*
		Male	Female		Academic	Clinical	Interns	
**Safety**, median (IQR) [mean rank]	8 (7–8)	8 (7–9) [595]	8 (7–8) [545]	**0.009**	7 (6–8) [500]	8 (7–9) [607]	8 (7–9) [613]	**< 0.001**
**Efficacy**, median (IQR) [mean rank]	8 (7–9)	8 (7–9) [577]	8 (7–9) [565]	**0.521**	7 (6–8) [500]	8 (7–9) [601]	8 (7–9) [620]	**< 0.001**
**Hesitancy**, median (IQR) [mean rank]	5 (2–7)	5 (2–7) [570]	5 (2–7) [572]	**0.893**	5 (3–7) [574]	5 (2–7) [582]	5 (2–7) [554]	**0.495**

* Mann Whitney test was used

** Kruskal Wallis test was used

### Factors affecting students’ opinions towards booster vaccination

**Fig. 2:**
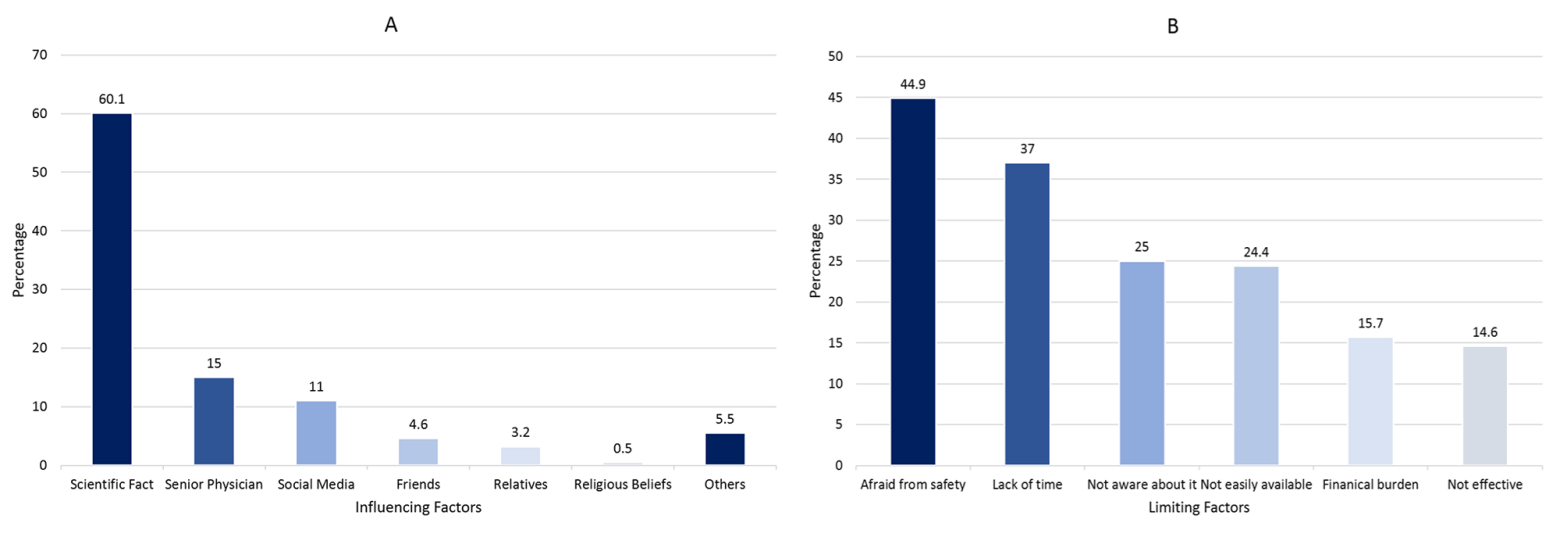
Factors influencing (**A**) and limitations (**B**) of vaccination with HCWs? recommended vaccines

The most influential factors in students’ opinions regarding the booster vaccination were scientific facts (60.1%) and senior physicians or professors (15%) (Fig. 2A). There was a significant association between the factors affecting students’ opinions and gender (*P* = 0.01), educational grade (*P* = 0.01), and nationality (*P* = 0.002). Academic students were more likely to be influenced by social media than interns (14.6% vs. 7.8%, *P* = 0.01). However, there was no significant association with other demographic factors (*P* > 0.05).

### Limitations of receiving booster doses

The main factors that prevented students from receiving vaccines were fear of vaccine side effects (44.9%) and lack of time (37%) (Fig. 2B). There was a significant association between students who chose fear of vaccine effects and gender (female: 51.9% vs. male: 38.3%; *P* < 0.0001), educational level (academic: 52.9% vs. clinical: 44.8% vs. interns: 35.6%; *P* < 0.0001), and residence type (urban: 47.1% vs. rural: 41%; *P* = 0.0447). Regarding students who cited a lack of time, there was a significant association with gender (males: 40% vs. females: 33.7%; *P* = 0.0268), educational grade (interns: 41.9% vs. clinical: 40.9% vs. academic: 29%; *P* = 0.0002), and nationality (Egyptian: 38.3% vs. non-Egyptian: 25.6%; *P* = 0.0062). Delta region (27.5%; *P* = 0.0126) was less likely to choose a lack of time. Participants residing in urban areas significantly reported lack of time (39.9% vs. 31.8%, *P* = 0.01) as the main factor limiting vaccination uptake, whereas rural residents reported high vaccination costs as the primary limitation (18.8% vs. 13.9%, *P* = 0.03).

### Flu vaccine

Approximately 35% of participants were aware that influenza vaccination is one of the HCW-recommended vaccinations. Regarding vaccination rates, 17.6% reported receiving a seasonal flu vaccine in the past year and approximately 49.4% reported having been vaccinated at least once before. However, only 5.1% of students reported receiving a booster vaccine every season. Gender (males: 31.3% vs. females: 21.9%, *P* = 0.002), generation (non-first-generation: 31.6% vs. first-generation: 24.2%, *P* = 0.002), and educational grade (academic: 35.9% vs. clinical: 21.5%; *P* = 0.001) were significantly associated with receiving the flu vaccine multiple times, but not seasonally. In contrast, the type of residence and region did not affect the rate of influenza vaccination (*P* > 0.05).

### COVID-19 vaccine

Notably, about 92% of the participants received at least one dose of COVID-19, and 79.4% received at least two doses of COVID-19 vaccine. Among those who received COVID-19 vaccination, the most commonly received COVID-19 vaccines were Oxford/AstraZeneca (27%) and Sinovac (25.9%). Regarding booster doses, 66.3% of our participants received at least one booster dose of the COVID-19 vaccine. Oxford/AstraZeneca (26%), Sinovac (25.4%), and Pfizer (21%) were the most commonly reported boosters. More than 75% of the participants expressed a willingness to receive a yearly booster if recommended. Gender (male: 60.5% vs. female: 39.5%; *P* = 0.028) and educational level (academic: 63.4% vs. clinical: 59.6% vs. interns: 47.4%; *P* < 0.001) were significantly associated with receiving two doses of COVID-19 vaccine (*P* = 0.03), whereas no significant associations were observed with other demographic factors.

## Discussion

To the best of our knowledge, this is the first study from Egypt to examine the knowledge, attitude, and practice of medical students towards HCWs’ recommended vaccines across various educational levels, including interns. Overall, there was a positive attitude towards HCWs’ recommended vaccination. However, most participants had moderate knowledge with a gap in the actual practice of receiving vaccines.

### Knowledge of HCWs’ recommended vaccines

Approximately 43% and 25% of the students showed moderate and high knowledge levels, respectively. Notably, knowledge increased with educational grade, particularly among interns and clinical-level students, compared to academic-year students. This difference in knowledge likely stems from the greater exposure of these students to clinical settings, which enables them to witness the impact of vaccination in real-world scenarios. Moreover, a more rigorous curriculum for clinical-level students encompasses comprehensive information about vaccinations, facilitating the accumulation of knowledge over time [20, 21]. These findings align with those of previous studies conducted in the United States and Europe [13, 22–24]. Similarly, gender showed a significant association with knowledge about HCWs’ recommended vaccines, with females being more likely to have higher knowledge than males which is inconsistent with previous studies from Europe that showed no difference [13].

### Practice of participants towards HCWs’ recommended vaccines

Our study found a notable disparity between medical students’ knowledge and practice, as only 8.1% of participants reported receiving two or three recommended vaccines in the past decade. Consistent with literature, HBV vaccine had the highest rates of both knowledge (70.9%) and practice (66.7%), likely due to the high prevalence of HBV infection in Egypt [25]. Previous studies have reported similar findings in Vienna, and Pakistan, where the rate of HBV vaccination reached 80% [26, 27]. Conversely, varicella and meningococcal vaccines are the least known and received vaccines. In our study, the MMR vaccination rate was 29.2%, which is lower than the average vaccination rates reported in several European countries which was about 80% [13].

Similar to knowledge, interns were more likely to receive booster vaccination which is consistent with findings from previous studies [13]. However, there was no significant difference in booster vaccination rates between academic- and clinical-year students or between male and female students. Having a vaccination center at the participants’ institutions or in their respective regions was positively correlated with increased knowledge and vaccine uptake. This underscores the potential of these centers to raise awareness and promote adherence to the recommended vaccines among healthcare students.

### Attitude of participants towards HCWs’ recommended vaccines

The participants expressed positive views on vaccine safety and effectiveness, with little variation. Consistent with the literature, the majority (91%) of participants believed that vaccines were safe and effective [26, 28]. Attitudes towards booster vaccines were also positive due to awareness of the need for revaccination. Medical training and clinical exposure influenced attitudes, with interns and clinical year students reporting higher scores. Although 90% of the participants agreed that vaccination was safe and that everyone should be vaccinated, only 74% would recommend booster vaccinations to their colleagues, friends, and family. This discrepancy may reflect vaccine hesitancy among the participants. Our study found a median hesitancy score of 5/10, with 7.2% of the participants expressing uncertainty or unwillingness to receive booster vaccines. Similar hesitancy patterns are observed among medical students regarding influenza, COVID-19, and HBV vaccines [13, 24–26].

### Factors that limit vaccine uptake

When participants were asked about reasons that might prevent them from receiving booster vaccinations, 44.9% reported their fear of side effects as a major concern. This fear could be influenced by misinformation regarding the side effects of COVID-19 vaccines that circulated just before this study was conducted [29, 30]. Interestingly, most of those who reported fear as a limitation were students in their academic years. Meanwhile, interns and students in their clinical years cited a lack of time as the main limitation. Additionally, participants from urban areas were more likely to cite lack of time as a limitation.

### Factors that influence vaccine uptake

The most influential factors shaping medical students’ opinions on vaccination were scientific facts (60.1%), and senior physicians or professors (15%). Interns (63%) and clinical students (62.7%) were more likely to form opinions based on scientific facts. Interestingly, participants who reported social media as an influencing factor had less positive attitudes towards vaccine safety and efficacy than those influenced by scientific facts or senior physicians. These observations highlight the need for evidence-based and accurate information regarding the dissemination of vaccines among healthcare students.

### Flu vaccine

Our study found that only 35% of the participants were aware that the flu vaccine is recommended for HCWs, ranking fourth on the list. Additionally, while 49.4% reported receiving at least one influenza vaccine shot, only 17.6% had received a shot within the previous year, which is similar to the results of a previous study [12]. Surprisingly, academic students were more likely to have received the seasonal flu vaccine in the past year than clinical students and interns, in contrast to the findings of Walker et al. [12] but consistent with Rostkowska et al. and Gray et al. [13, 31]. This may be due to academic students’ greater exposure to vaccination opportunities on university campuses and their compliance with vaccination requirements for attending classes or clinical placements [32].

### COVID-19 vaccine

Approximately 80% of the participants in our study received at least two doses of the COVID-19 vaccine, consistent with findings in Egypt, and nearly double the rate of the general population (44.3%) [16, 33]. This high vaccination rate may be attributed to the government’s requirement for all university students to be vaccinated before returning to campus, after the lockdown measures were lifted. Notably, this trend aligns with the higher coverage rates among healthcare workers in Germany (91.7%), Italy (82%), Portugal (87%), and Greece (81.9%). Similar to the pattern observed for influenza vaccination, academic students were more likely to receive the COVID-19 vaccine than clinical students and interns.

### Limitations of our study

Although our study has several strengths, including having participants from all study years in nine institutions, with a high response rate of 86.4%, some limitations should be considered. First, we were unable to collect data from the Fayoum geographic region, which represents less than 5% of all Egyptian medical students. Second, the timing of our questionnaire, conducted shortly after the onset of the COVID-19 pandemic, may have influenced some results, particularly the attitudes toward vaccination. Additionally, due to questionnaire length constraints, we could not include sections on other vaccines beyond flu and COVID-19, which were prioritized given the ongoing pandemic.

## Conclusions

Overall, medical students showed intermediate knowledge of HCWs’ recommended vaccines with a positive attitude towards vaccination. The participants expressed strong agreement on the safety and efficacy of the vaccines with neutral hesitancy. Additionally, the students supported the implementation of mandatory booster vaccines for both the staff and students. However, there is still a gap in vaccination practices. Therefore, we recommend initiating vaccination campaigns to raise awareness and enhance vaccination practices, particularly for medical education and training.

### Electronic supplementary material

Below is the link to the electronic supplementary material.

Supplementary Material 1

## Data Availability

The datasets used and/or analyzed during the current study are available from the corresponding author upon reasonable request.

## Abbreviations

CDC: Center for Disease Control
COVID-19: Coronavirus Disease of 2019
DTaP: Diphtheria, Tetanus, Pertussis
HBV: Hepatitis B Virus
HCWs: Healthcare care workers
IQR: Interquartile range
MMR: Measles, Mumps, Rubella
WHO: World Health Organization
