# Supplementary material for: Knowledge, attitude, and practice of Egyptian medical students towards healthcare workers’ recommended vaccines: a nationwide cross-sectional survey
Source: BMC Med Educ. 2024 Aug 14;24:876. doi: 10.1186/s12909-024-05712-8 (PMC11323588; doi:10.1186/s12909-024-05712-8)
Supplement: Supplementary file 1 — Supplementary Material 1 [file 12909_2024_5712_MOESM1_ESM.docx]

# Supplement 1: Study questionnaire

# Consent

You are invited to participate in a research study about the prevalence and attitude of medical students towards recommended booster vaccinations among healthcare workers.

This survey will take approximately about 1-5 minutes of your time. You will be asked to complete an online survey about the prevalence and attitude of medical students towards recommended booster vaccinations among healthcare workers.

Your decision to participate or decline participation in this study is completely voluntary and you have the right to terminate your participation at any time without penalty. If you want do not wish to complete this survey just close your browser.

Your participation in this research will be completely confidential and data will be averaged and reported in aggregate without any personal data in the statistical analysis.

Although your participation in this research may not benefit you personally, it will help us to estimate the the prevalence and attitude of medical students towards recommended booster vaccinations among healthcare workers in the Egyptian medical schools as a representative of one of the developing countries to evaluate this experience on this important part of the world on community based results. There are no risks to individuals participating in this survey beyond those that exist in daily life.

If you have questions about this project, you may contact us on: [mohammedshowkey1997@gmail.com](mailto:mohammedshowkey1997@gmail.com)

**"Consent"**

I have read and understand the above consent form, I certify that I am 18 years old or older and, by clicking the submit button to enter the survey, I indicate my willingness voluntarily take part in the study.

**-Do you accept sharing in this Study?**

1. Yes
2. No

**-Please, write your code: ..........**

**-Please, write your name to ensure that you are the included participant (your name will never be used in the analysis and will be kept confidential): ......**

**Section (1): Socio-demographics:**

1. **Sex:**
   1. Male
   2. Female
2. **Age: .........**
3. **What is your educational grade: (Dropdown list)**
4. First grade
5. Second grade
6. Third grade
7. Fourth grade
8. Fifth grade
9. Sixth grade
10. House officer
11. **You are a:**
12. First generation medical student (you are the first one in your family to join medical school)
13. Not a first generation medical student (you have other family members joined medical school before)
14. **What is your current governorate you are living in: (Dropdown list)**
15. Cairo
16. Giza
17. Qalyubia
18. Alexandria
19. Matruh
20. Beheira
21. Damietta
22. Menoufia
23. Gharbia
24. Kafer Elshikh
25. Dakahlia
26. Sharqia
27. Ismailia
28. Suez
29. Port Said
30. North Sinai
31. South Sinai
32. Minya
33. Bani Suef
34. Faiyum
35. Asyut
36. New Valley
37. Sohag
38. Qina
39. Luxor
40. Aswan
41. Red sea
42. **What is the type of your current residency**:
43. Rural
44. Urban
45. **Marital status:**
46. Single
47. Married
48. Divorced
49. **Nationality:**
50. Egyptian
51. Not Egyptian
52. **Is there available vaccination center in your institution/governorate where you can take voluntary vaccines?**
53. Yes
54. No
55. I am not sure

**Section (2): knowledge and practice of booster vaccination:**

- - - 1. **Did you get vaccinated as part of the National Vaccination Program of your country during infancy?**

| 1. Yes |
| --- |
| 1. No |
| 1. I don't know |

- - - 1. **Which of the following vaccines booster are recommended by the World Health Organization (WHO) and Center of Disease Control (CDC) for medical staff?**

1. Diphtheria, tetanus, Pertussis
2. Measles, mumps and rubella
3. Hepatitis B
4. Pneumococcal pneumonia
5. Meningococcus invasive infection
6. Human papillomavirus (condylomas, cervical cancer)
7. Influenza
8. COVID-19
9. *Hemophilus influenza b* meningitis
10. Shingles
11. I don’t know
    - - 1. **For which of the following vaccines you have had a booster dose for in the last 10 years?**
12. Yes, for all vaccines
13. Yes, for some vaccines:

Diphtheria, tetanus, pertussis

Measles, mumps and rubella

Hepatitis B

Pneumococcal pneumonia

 Meningococcus invasive infection

Human papillomavirus (condylomas, cervical cancer)

Influenza

COVID-19

*Haemophilus influenza b* meningitis

 Shingles

1. I did not take any booster doses
2. I don’t know

**Section (3): Attitude toward vaccinations:**

1. **Which statement describes your opinion about vaccinations the most?**

| 1. It is useful and safe and I think that everybody should get vaccinated. |
| --- |
| 1. There is too little evidence to prove that it is effective. |
| 1. There is too little evidence to prove that it is even safe 2. It is neither effective nor safe. |

1. **Do you know that in order to be protected properly you need to get revaccinated for several vaccines?**
2. Yes, I am aware it and doing it properly.
3. Yes, I am aware of it, but I am not sure if I have full vaccination.
4. No, this is the first time I hear about that.
5. No, there is no need because vaccination is always life-long protection
6. **Do you intend to take booster doses of HCWs' recommended vaccines or have a blood test to ensure immunity?**
7. Yes
8. No
9. Not sure
10. **Do you think that booster vaccination for the recommended vaccines for medical personals should be mandatory for medical staff (attending doctors, nurses etc.)?**

| 1. Yes 2. No 3. No sure |
| --- |

1. **Do you think that booster vaccination for the recommended vaccines for medical personals should be mandatory for medical students?**

| 1. Yes 2. No 3. Not sure |
| --- |

1. **What influences your opinion about booster vaccinations the most?**

| 1. Scientfic facts |
| --- |
| 1. Social Media |
| 1. Senior physicians, professors |
| 1. My relatives |
| 1. Religious beliefs |
| 1. My friends, colleagues 2. Others (please mention |

1. **Do you recommend your relatives, friends, colleagues etc. to take a booster dose of vaccines?**

| 1. Yes |
| --- |
| 1. No |
| 1. Never thought about that |

1. **In general, how do you evaluate booster vaccine safety on a 0-10 scale?**

(0 for “Booster vaccination is not safe” and 10 for “Booster vaccination is safe”)

1. **In general, how do you evaluate booster vaccine efficacy on a 0-10 scale?**

(0 for “Booster vaccination is not effective” and 10 for “Booster vaccination is effective”)

1. **How do you evaluate your own booster vaccine hesitancy on a 0-10 scale?**

(0 for “no hesitancy regarding vaccination” and 10 for “maximal hesitancy”)

1. **Which of the following may prevent you taking booster vaccination?**
2. Not aware about it
3. Lack of time
4. High vaccine cost
5. Not important or effective
6. Vaccine is not easily available
7. Afraid from vaccine side effects or safety

**Section (4): Attitude toward specific vaccines:**

- - 1. **FLU VACCINE**

1. **Did you get seasonal flu vaccine on the last year?**
2. Yes
3. No
4. **How often do you get vaccinated against seasonal flu?**

| 1. Every other season. |
| --- |
| 1. Every season. |
| 1. I have never been vaccinated against seasonal flu. |
| 1. I have only been vaccinated once. |
| 1. Not regularly. |

**B) COVID VACCINE**

1. **If you get COVID-19 vaccine, how many COVID-19 vaccine doses did you get?**
2. One dose
3. Two doses
4. Three doses
5. Four doses
6. **Which type of vaccine did you get for the main vaccine (first one/two doses)?**
7. Janssen
8. Pfizer
9. Moderna
10. Oxford/Astrazeneca
11. Sinopharm
12. Sinovac
13. Bharat
14. Novavax
15. **Which type of booster dose (second/third/fourth doses) did you get?**

1. Janssen

2. Pfizer

3. Moderna

4. Oxford/Astrazeneca

5. Sinopharm

6. Sinovac

7. Bharat

8. Novavax

1. **If a yearly COVID-19 booster vaccine dose became recommended for healthcare workers, will you take it in the future**
2. Yes
3. No
4. I am not sure

# Supplement 2: Sampling strategy

#

Cairo region

- Cairo
- Benha
- Helwan

Alexandria region

- Alexandria

Delta region

- Menoufia
- Damietta branch of Al-Azhar

We have 47 faculties of medicine in Egypt

26 faculties met our inclusion criteria

10 faculties were randomly selected according to the weight of facilities in each region

Canal region

- Zagazig

North Upper Egypt

- Fayoum

Assuit

- Assuit branch of Al-Azhar

South Upper Egypt

- South Vally

# Supplement 3: Sampling distribution

We could reach 1321

11 could not be reached

180 do not respond

1141 responded to our survey

Sample was calculated to be 1024

Total sample was 1332

30% non-response rate

- Divided according to the weight of each faculty.
- Participants were randomly selected.

Cairo

299

Alexandria

282

Benha

121

Helwan

86

Menoufia

166

Damietta

45

Zagazig

233

Assuit

45

South Valley

55
